# Circulating MicroRNAs in Serum from Cattle Challenged with Bovine Viral Diarrhea Virus^‡^

**DOI:** 10.3389/fgene.2017.00091

**Published:** 2017-06-28

**Authors:** Tasia M. Taxis, Fernando V. Bauermann, Julia F. Ridpath, Eduardo Casas

**Affiliations:** Ruminant Diseases and Immunology Research Unit, National Animal Disease Center, United States Department of Agriculture – Agricultural Research Service, AmesIA, United States

**Keywords:** BVDV, cattle, microRNA, miR-423-5p, miR-151-3p, *Pestivirus*, serum

## Abstract

Bovine viral diarrhea virus (BVDV) is an RNA virus that is often associated with respiratory disease in cattle. MicroRNAs have been proposed as indicators of exposure to respiratory pathogens. The objective of this study was to identify microRNAs in cattle that had been challenged with a non-cytopathic field strain of BVDV. Five colostrum deprived neonate Holstein calves were inoculated with BVDV (challenged) and 4 were mock challenged (control). Serum from all calves was collected at four different times: prior to challenge (day 0) and at 4, 9, and 16 days post-challenge. RNA was extracted from sera, and expression, via read counts, of small non-coding RNAs were obtained using next-generation sequencing. A total of 905,861 sequences identified 427 microRNAs. Sixty-two microRNAs had >1,000 total reads across all samples. Bta-miR-339a, bta-miR-185, bta-miR-486, Bta-miR-92a, bta-miR-30e-5p, bta-let-7c, and bta-miR-2284x were significantly different (*P* < 0.05) across time regardless of challenge status. Bta-miR-423-5p (*P* = 0.008) and bta-miR-151-3p (*P* = 0.005) were significantly different between challenged and control animals across time. In challenged animals, bta-miR-423-5p peaked in number of reads by day 4 and steadily declined from day 4 to day 16. In control animals, bta-miR-423-5p declined from day 0 to day 9 and increased in number by day 16. By day 16, both challenged and control animals had similar levels of bta-miR-423-5p, and these levels were similar to day 0 levels. Bta-miR-151-3p peaked at day 9 in challenged animals, while control animals decreased across time. By day 16, the number of reads of bta-miR-151-3p were similar between challenged and control animals. The level in challenged animals had returned to day 0 levels by day 16, whereas the levels for control animals was significantly lower (*P* = 0.006) than day 0. Further studies are needed to establish if bta-miR-423-5p or bta-miR-151-3p could be used as a biomarker for exposure to BVDV.

## Introduction

Bovine viral diarrhea viruses (BVDV) comprise two species within the *Pestivirus* genus that cause disease in ruminants. Producers worldwide experience financial loss due to BVDV infections (Houe, 2003; Ridpath and Fulton, 2009). Animals with BVDV can exhibit several clinical signs including diarrhea, depression, and pyrexia ranging from clinically mild to severe; however, in many cases, signs of BVDV infections are subclinical and go undiagnosed or unnoticed (Houe, 2003; Liebler-Tenorio et al., 2004; Ridpath and Fulton, 2009; Walz et al., 2010). Regardless of clinical presentation, all BVDV infections result in significant loss of immune tissue, and it is theorized that the resulting immune suppression potentiates increased severity of subsequent infections (Roth et al., 1981, 1986; Roth and Kaeberle, 1983; Brown et al., 1991). Vaccine combinations are available to limit transmission and clinical disease, but not necessarily prevent BVDV infections (Fulton et al., 2003; Ridpath and Fulton, 2009; Walz et al., 2010). Identifying animals that may have damaged immune systems and that are at risk to develop respiratory disease could improve herd health management.

Before the identification and characterization of lin-4 in 1993, research on microRNAs was non-existent (Wahid et al., 2010). MicroRNAs, are small (21–25 nucleotides) single-stranded non-coding RNAs that have been shown to regulate gene expression post-transcriptionally (Wahid et al., 2010; Dozmorov et al., 2013; Lai et al., 2013; Kim et al., 2015). They have been associated with many biological processes including development, innate immunity, adaptive immune response, and disease (Wahid et al., 2010; Lai et al., 2013; Li et al., 2013; Hitachi et al., 2014; Chen R.J. et al., 2015; Chen Z. et al., 2015; Casas et al., 2016).

Studies that identify microRNAs associated with cattle acutely infected with BVDV are non-existent. The type of tissue utilized in such a study should be applicable to the study’s objective. The objective of this study was to identify microRNAs found in the serum of colostrum deprived Holstein calves that are associated with infection from a non-cytopathic field strain of BVDV of typical virulence (Liebler-Tenorio et al., 2004). Results from this study will create knowledge of dysregulated microRNAs to possibly identify biomarkers of a BVDV infection. Ideally, the sampled tissue should be able to be collected with minimal harm to the animal. Sera was utilized in the current study.

## Materials and Methods

The animals used in this study represented positive and control groups within two larger studies. Animals were housed and cared for according to the management protocol approved by the Animal Care and Use Committee of the National Animal Disease Center in Ames, IA, United States (ARS-2667).

### Virus Characterization and Propagation

The BVDV2 strain used in these studies, BVDV2-RS886, was first isolated from a persistently infected calf (Liebler-Tenorio et al., 2004). Under controlled conditions, infection of colostrum deprived calves with this virus strain results in a low-grade pyrexia and leukopenia, or a reduction in circulating immune cells (Ridpath et al., 2013). Virus was propagated and inoculum prepared as described previously (Ridpath et al., 2013).

### Animals

In two replicate studies a total of nine colostrum deprived male Holstein calves between 3 and 5 weeks of age were assigned to either a control or challenge group. In both replicates of the study, calves in the control group (*n* = 4) were mock-inoculated on day 0 with lysed cell culture supernatant from non-infected Madin Darby bovine kidney (MDBK) cells. Calves in the BVDV challenge group (*n* = 5) were exposed to a non-cytopathic field strain of BVDV (BVDV2-RS886) on day 0. Inoculations consisted of 4 mL of viral preparation (1 × 10^6^ TCID/mL) or clarified freeze/thaw lysate of MDBK cells and was delivered by the nasal route using either an intranasal atomization (LMA MAD300 Nasal atomizer, Teleflex, Inc., Research Triangle Park, NC, United States) or by placing directly in the nose.

Following inoculation calves were observed twice daily for signs of respiratory disease. Fresh serum samples were collected at four different times; (1) a baseline measurement (day 0); collected prior to the initial inoculation; (2) 4 days post-initial inoculation; (3) 9 days post-initial inoculation; and (4) 16 days post-initial inoculation. Serum samples were collected from all nine calves at all four times via jugular venipuncture in SST vacutainer tubes (BD, Franklin Lakes, NJ, United States). The vacutainer tubes were incubated at 37°C for 30 min. and were centrifuged at 1250 × *g* for 30 min. The isolated serum samples were placed into 2 mL vials and were stored at -80°C until processed.

### MicroRNA Isolation

Purification of total RNA from the serum samples was completed using the miRNeasy Serum/Plasma Kit (QIAGEN, Germantown, MD, United States). The RNA was extracted according to manufacturer’s instructions, and the samples were eluted in 14 μL RNase-free water. After extraction, the concentration of microRNAs extracted in each sample was determined using a 10–40 nucleotide gate on an Agilent 2100 Bioanalyzer Small RNA chip (Agilent Technologies, Santa Clara, CA, United States).

### Library Preparation and Sequencing

Libraries were prepared using the NEBNext Multiplex Small RNA Library Prep Set for Illumina Set 1 and 2 (New England BioLabs, Ipswich, MA, United States). Six microliters (6 μL) of each extracted serum sample was individually indexed with one of the Illumina 1-23 indexed primers. Library PCR products were then purified to single- and double-stranded DNA fragments and concentrated to 35 μL using the QIAquick PCR purification kit (QIAGEN, Germantown, MD, United States). The quality and quantity of each library was determined using a 135–170 nucleotide gate on an Agilent 2100 Bioanalyzer High Sensitivity DNA chip (Agilent Technologies, Santa Clara, CA, United States). A total of 30 ng of each library was pooled into one of two pools. Twenty-three total libraries in each pool were created and size selected (142–170 nt) according to manufacturer’s instructions using the Pippin Prep on a 3% Agarose gel without Ethidium Bromide (SAGE Sciences, Beverly, MA, United States). After the gel was run, the pools were concentrated using the QIAquick PCR purification kit (QIAGEN, Germantown, MD, United States) by eluting in 32 μL of RNase-free water. The concentration of each pool was determined using a 135–170 nucleotide gate on an Agilent 2100 Bioanalyzer High Sensitivity DNA chip (Agilent Technologies, Santa Clara, CA, United States). The pool was stored at -20°C until sequencing. Each pool was sequenced by 50 cycles on the Illumina HiSeq 2500 System (Illumina, San Diego, CA, United States).

### Data and Statistical Analysis

The Illumina sequences were inspected for quality using FastQC v0.11.2^1^, and the adapter was removed using the fastx_clipper program in a fastx toolkit^2^. Sequences of bovine microRNAs and their precursors were downloaded from miRBase (v21^3^). Reads were mapped to known bovine microRNAs, and read counts for each microRNA were compiled and normalized using miRDeep2 (Friedlander et al., 2012). In order to obtain minimum statistical power, analysis was completed on microRNAs with >1,000 total reads (Wilson Van Voorhis and Morgan, 2007; Motameny et al., 2010). Sequences are available on the NCBI SRA under BioProject accession SRP091488.

Statistical analysis was completed using the Mixed procedure of SAS (SAS, Inst. Inc., Cary, NC, United States). The model included the main effects of treatment (challenge or control groups), time (days 0, 4, 9, and 16), and the interaction between treatment and time. Day 0 read counts were included in the model as a covariate, the replicate of the study (1 or 2) was included as a random effect, and animal ID was included as a repeated measure. The study was designed to ascertain nominal significant differences with the minimal number of animals; therefore, un-corrected significances are presented. Significances should be taken in consideration when interpreting results.

## Results

At day 3 all BVDV challenged animals started to develop fever and lymphopenia. The fevers resolved by day 7 and lymphocyte counts started to increase by day 9; however, lymphocyte counts remained higher than pre-challenge levels at day 16. Control animals showed no signs of fever or lymphopenia.

A total of 191,071,075 reads were obtained from sequencing, and 905,861 reads mapped to bovine microRNAs. Four hundred twenty-seven different microRNAs were identified with at least one read among all animals. Of which, 288 microRNAs had <100 total reads and 77 microRNAs had between 100 and 1,000 total reads. Sixty-two microRNAs had greater than 1,000 total reads.

Those 62 microRNAs were further analyzed (Supplementary Table 1). Mean counts between BVDV challenged and control animals were similar for all 62 microRNAs (*P* > 0.05).

Seven microRNAs were significantly different across time, regardless of treatment group. **Table 1** shows the normalized mean read counts across all animals, regardless of treatment, for each significant microRNA across time. Between day 0 and day 4, two microRNAs increased in number of reads, two decreased, and three remained statistically constant during this period. Between day 4 and day 9, three microRNAs increased in number of reads, while four remained constant. Between day 9 and day 16, six microRNAs decreased in number of reads, and only bta-miR-185 remained constant during this period.

**Table 1:** MicroRNA, normalized mean in reads per million (RPM) for each response time, standard error (SE), and their association (*P*-value) across all animals.

	Response time point			
microRNA	Day 0^1^	Day 4	Day 9	Day 16	SE	*P*-value
Bta-miR-339a	902^a^	1,526^a^	2,504^b^	800^a^	319	0.003
Bta-let-7c	1,952^a^	908^b^	1,498^a^	887^b^	202	0.002
Bta-miR-486	40,417^a,c^	86,065^a,b^	103,779^b^	35,799^c^	17,744	0.01
Bta-miR-2284x	2,208^a^	1,421^b^	2,113^a^	1,542^b^	165	0.02
Bta-miR-30e-5p	1,462^a^	2,972^b^	2,901^b^	2,781^b^	476	0.02
Bta-miR-185	683^a^	1,233^a,b^	1,583^a^	633^b^	357	0.03
Bta-miR-92a	24,695^a^	40,525^b,c^	46,815^b^	28,725^a,c^	7,079	0.03

*^1^Day 0 samples were collected 1–2 days prior to challenge. ^a,b,c^Means (RPM) without a common superscript within row are statistically different (*P* ≤ 0.05).*

**FIGURE 1:**
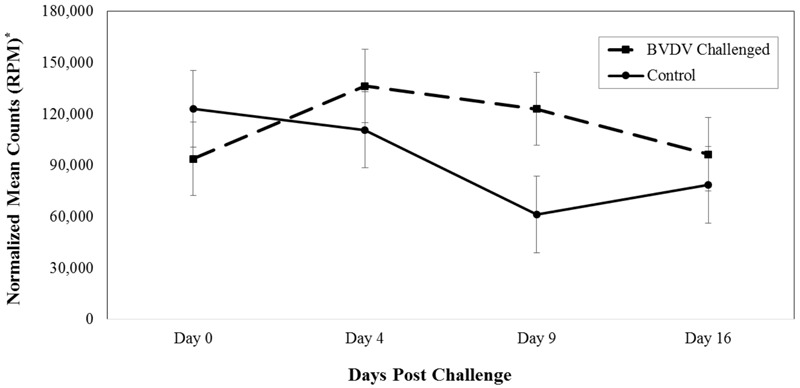
Interaction of time and challenge status for bta-miR-423-5p (*P* = 0.008). ^∗^RPM stands for reads per million. Dashed line corresponds to cattle challenged with bovine viral diarrhea virus (BVDV). Solid line corresponds to control animals.

Bta-miR-423-5p (*P* = 0.008) differed in number of reads between BVDV challenged and control animals across time. The number of reads for bta-miR-423-5p in BVDV challenged animals peaked above day 0 levels by day 4 then decreased over time while the number of reads in control animals tended to decrease throughout the study (**Figure 1**). Significant differences between BVDV challenged and control animals at individual time points were undetected, however, levels of bta-miR-423-5p in both BVDV challenged and control groups returned to day 0 levels by day 16.

**FIGURE 2:**
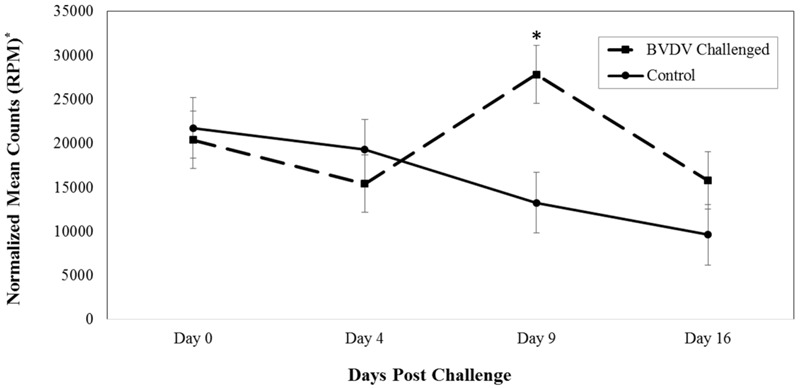
Interaction of time and challenge status for bta-miR-151-3p (*P* = 0.005). ^∗^RPM stands for reads per million. Dashed line corresponds to cattle challenged with BVDV. Solid line corresponds to control animals. An asterisk above a time point represents a significant (*P* < 0.05) difference in number of read counts between BVDV challenged and control animals at that particular time point.

Bta-miR-151-3p (*P* = 0.005) also differed in number of reads between BVDV challenged and control animals across time. The number of reads for bta-miR-151-3p in BVDV challenged animals peaked above day 0 levels by day 9 and returned to day 0 levels by day 16 (**Figure 2**). In control animals, the number of reads for bta-miR-151-3p steadily decreased across time, resulting in significantly lower levels from day 0 levels by day 16 (*P* = 0.007). Nine days after being challenged, BVDV challenged animals had a significantly greater number of reads of bta-miR-151-3p than control animals (*P* = 0.006).

## Discussion

MiR-151-3p was shown to be significantly lower in cells that were infected with *Brucella melitensis* (Zheng et al., 2012). *B. melitensis* replicates within host cells while remaining undetected from the host immune system (Zheng et al., 2012; Lacerda et al., 2013). Some studies indicate that host microRNA expression can be regulated by pathogens (Cameron et al., 2008; Cremer et al., 2009, 2012; Zheng et al., 2012). Unlike *B. melitensis*, BVDV does not avoid a host immune response to survive. Potentially then, *B. melitensis* infected cells express a lower level of miR-151-3p in order to avoid a host immune response, while BVDV, which does elicit a host immune response, showed an increase in miR-151-3p, particularly 9 days after infection. The differences in expression of miR-151-3p between *Brucella melitensis* and BVDV infection could be due to the differences in the way the pathogens replicate in the host.

MiR-423-5p has been identified in humans and cattle with disease and viral or bacterial infections (Lagatie et al., 2013; Brown et al., 2014; Jin et al., 2014; Lawless et al., 2014; Punga et al., 2016). An increase of miR-423-5p was observed in humans that had myasthenia gravis, pulmonary fibrosis, or were infected with a polyomavirus, which is a non-enveloped DNA virus (Lagatie et al., 2013; Brown et al., 2014; Punga et al., 2016). In cattle, a similar up-regulation of miR-423-5p was seen in mammary glands exposed to *Streptococcus uberis* or *Escherichia coli* (Jin et al., 2014; Lawless et al., 2014). This study observed an increase of bta-miR-423-5p in BVDV challenged animals 4 days post-viral challenge. Additionally, bta-miR-423-5p in BVDV challenged animals tended to have a higher number of reads than control animals from day 4 through 16. Another study identified bta-miR-423-5p to be down-regulated in cattle exposed to *Mycoplasma bovis* vs. non-exposed cattle (Casas et al., 2016). It is possible that bta-miR-423-5p continues to decrease in levels after day 16, falling below the number of reads in control animals. The mature steers from Casas et al. (2016) study may have been exposed to *M. bovis* greater than 16 days prior to serum collection, supporting the lower expression levels of bta-miR-423-5p in positive animals.

Further research is needed to identify potential biomarkers to indicate timing of BVDV exposure in cattle. In this study, the most dramatic difference in number of reads between BVDV challenged and control animals was 9 days after BVDV exposure for both bta-miR-423-5p and bta-miR-151-3p. Four days after BVDV exposure, bta-miR-423-5p increased, with the number of reads higher than control animals, and bta-miR-151-3p remained similar to the control animals until day 9. Bta-miR-423-5p decreased in number of reads to become similar to control animals, while bta-miR-151-3p continued to have a higher number of reads in BVDV challenged animals than control animals on day 16. If bta-miR-423-5p or bta-miR-151-3p was to be used as a “BVDV timing” biomarker, results from this study suggest that serum collected 9 days post-BVDV challenge would be the most beneficial in order to differ between BVDV challenged and unchallenged animals. However, 9 day post-challenge, or infection may not be the most valuable time to identify the onset of respiratory disease.

Respiratory disease caused by BVDV results in an estimated $10-40 million loss per million calvings nationally (Houe, 2003). The clinical signs of cattle exposed to BVDV are diverse and most times undetectable, leading to difficulties in diagnoses or proper treatment (Fulton et al., 2003; Liebler-Tenorio et al., 2004; Walz et al., 2010). Once an animal is infected with BVDV, it is immune compromised, making the animal more at risk to develop respiratory disease. This study was conducted to identify potential microRNAs that could act as biomarkers indicating the timing of BVDV exposure to cattle. While there weren’t any microRNAs identified in this study that could be used as specific biomarkers of BVDV infection vs. non-infected cattle post-exposure, there were two microRNAs that could potentially be used as a biomarkers to indicate the timing of BVDV exposure.

## Disclosure

This research is part of an intramural project of the USDA/ARS National Animal Disease Center. The Federal Government had no role in the design, data collection and analysis, of the study; nor in the decision to prepare and publish the manuscript.

## Author Contributions

TT, FB, JR, and EC conceived the project and interpreted the results. FB, JR, and EC performed the experiment. TT wrote, and FB, JR, and EC reviewed the manuscript.

## Conflict of Interest Statement

The authors declare that the research was conducted in the absence of any commercial or financial relationships that could be construed as a potential conflict of interest. The reviewer RM and handling Editor declared their shared affiliation, and the handling Editor states that the process nevertheless met the standards of a fair and objective review.

## Footnotes

1 http://www.bioinformatics.babraham.ac.uk/projects/fastqc

2 http://hannonlab.cshl.edu/fastx_toolkit/

3 www.mirbase.org
