# Supplementary material for: Circulating MicroRNAs in Serum from Cattle Challenged with Bovine Viral Diarrhea Virus
Source: Front Genet. 2017 Jun 28;8:91. doi: 10.3389/fgene.2017.00091 (PMC5487392; doi:10.3389/fgene.2017.00091)
Supplement: Supplementary file 1 [file Table_1.DOCX]

| Supplementary Table 1. Total number of copies for each microRNA in the study. | |
| --- | --- |
| **microRNA** | **Number of copies** |
| bta-miR-185 | 1,030 |
| bta-miR-193a-5p | 1,031 |
| bta-miR-126-3p | 1,120 |
| bta-let-7c | 1,135 |
| bta-miR-760-3p | 1,154 |
| bta-miR-194 | 1,156 |
| bta-miR-148b | 1,249 |
| bta-miR-330 | 1,290 |
| bta-miR-339b | 1,317 |
| bta-miR-93 | 1,325 |
| bta-miR-339a | 1,450 |
| bta-miR-143 | 1,456 |
| bta-miR-150 | 1,507 |
| bta-miR-186 | 1,582 |
| bta-miR-10a | 1,616 |
| bta-miR-2284x | 1,674 |
| bta-miR-3432a | 1,685 |
| bta-miR-101 | 1,790 |
| bta-miR-30a-5p | 1,888 |
| bta-miR-222 | 1,969 |
| bta-miR-199a-3p | 1,972 |
| bta-miR-532 | 2,131 |
| bta-miR-215 | 2,137 |
| bta-miR-375 | 2,190 |
| bta-miR-142-5p | 2,221 |
| bta-miR-30e-5p | 2,305 |
| bta-miR-27a-3p | 2,572 |
| bta-let-7g | 2,828 |
| bta-miR-23a | 3,070 |
| bta-miR-221 | 3,436 |
| bta-miR-27b | 3,616 |
| bta-miR-378 | 3,657 |
| bta-miR-10b | 3,795 |
| bta-miR-1307 | 4,108 |
| bta-let-7f | 4,155 |
| bta-miR-181a | 5,102 |
| bta-let-7i | 5,440 |
| bta-miR-1246 | 7,147 |
| bta-miR-6529a | 7,438 |
| bta-miR-22-3p | 7,641 |
| bta-miR-223 | 8,353 |
| bta-let-7b | 9,262 |
| bta-miR-423-3p | 10,942 |
| bta-miR-191 | 11,184 |
| bta-miR-26a | 11,435 |
| bta-let-7a-5p | 11,575 |
| bta-miR-192 | 11,924 |
| bta-miR-99a-5p | 14,787 |
| bta-miR-151-3p | 14,908 |
| bta-miR-21-5p | 16,517 |
| bta-miR-451 | 19,732 |
| bta-miR-320a | 20,368 |
| bta-miR-24-3p | 22,825 |
| bta-miR-140 | 26,825 |
| bta-miR-30d | 27,056 |
| bta-miR-92a | 28,097 |
| bta-miR-128 | 29,137 |
| bta-miR-25 | 34,282 |
| bta-miR-486 | 56,030 |
| bta-miR-423-5p | 81,209 |
| bta-miR-148a | 103,083 |
| bta-miR-122 | 197,159 |
|  | |
|  | |
